# Supplementary figures and images for: Exploring COVID-19 pandemic perceptions and vaccine uptake among community members and primary healthcare workers in Nigeria: A mixed methods study
Source: PLoS One. 2026 Mar 11;21(3):e0310437. doi: 10.1371/journal.pone.0310437 (PMC12978461; doi:10.1371/journal.pone.0310437)

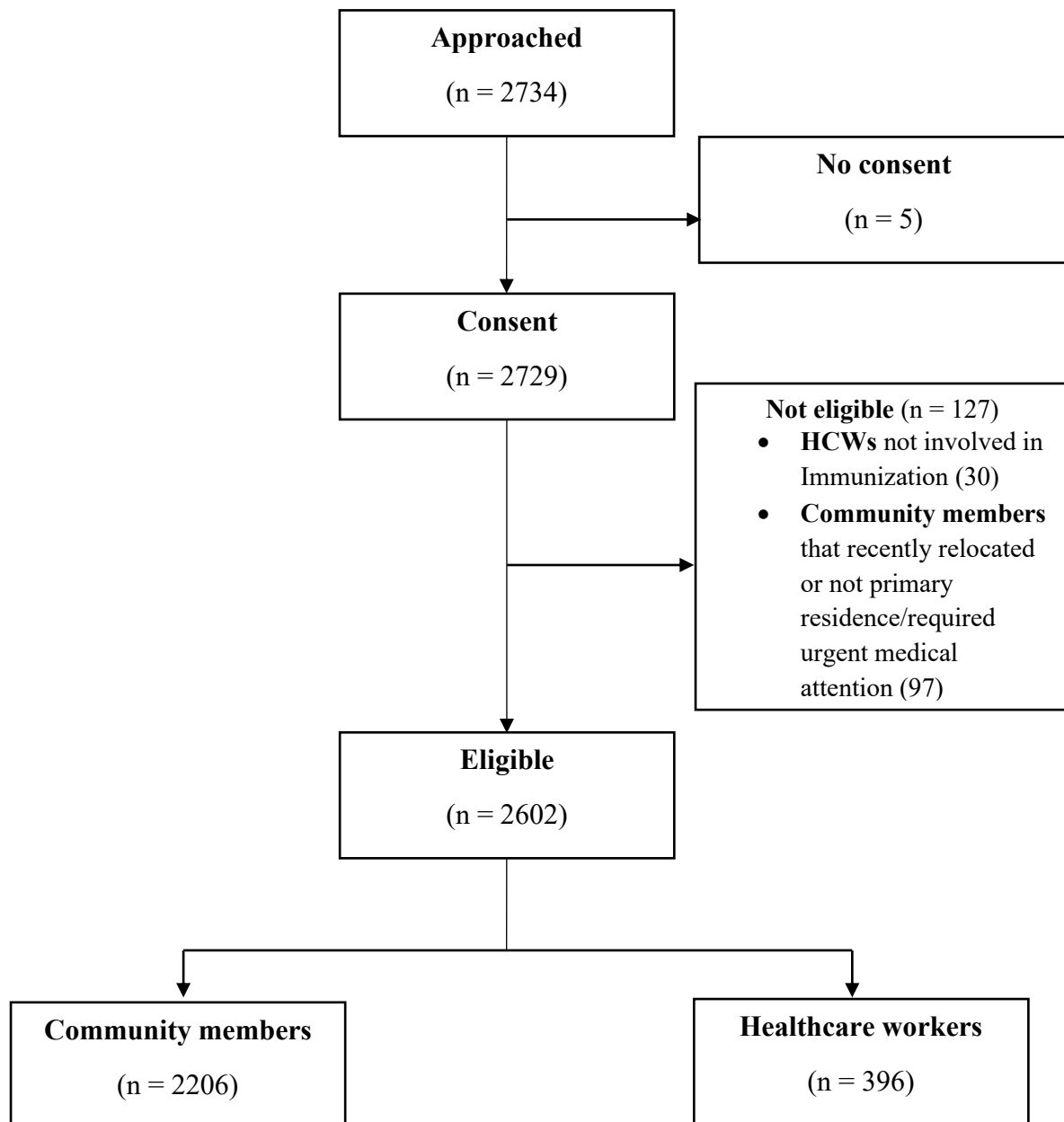

Supplement: S1 Fig — (PDF) [file pone.0310437.s001.pdf]
