# Supplementary material for: Exploring COVID-19 pandemic perceptions and vaccine uptake among community members and primary healthcare workers in Nigeria: A mixed methods study
Source: PLoS One. 2026 Mar 11;21(3):e0310437. doi: 10.1371/journal.pone.0310437 (PMC12978461; doi:10.1371/journal.pone.0310437)
Supplement: S1 Table — (PDF) [file pone.0310437.s002.pdf]

Participants characteristics	Community Members		Healthcare providers	
	Oyo (n=8)	Jigawa (n=8)	Oyo (n=6)	Jigawa (n=8)
**Sex**				
Male	2	1	0	6
Female	6	7	6	2
**Religion**				
Islam	5	8	3	8
Christianity	3	0	3	0
**Level of education**				
Primary/no formal	0	4	0	0
Secondary	2	3	1	0
Tertiary	6	0	5	8
