# Supplementary material for: Exploring COVID-19 pandemic perceptions and vaccine uptake among community members and primary healthcare workers in Nigeria: A mixed methods study
Source: PLoS One. 2026 Mar 11;21(3):e0310437. doi: 10.1371/journal.pone.0310437 (PMC12978461; doi:10.1371/journal.pone.0310437)
Supplement: S2 Table — a Respondents could provide multiple responses, bExcluding respondents that are not eligible for the dose, dose not required or did not indicate reasons. (PDF) [file pone.0310437.s003.pdf]

Reasons for not taking vaccine[a]	No dose[b]		No second dose[b]		No booster dose[b]	
	Community members (n = 1234)	Healthcare workers (n =13)	Community members (n =233)	Healthcare workers (n =23)	Community members (n=174)	Healthcare workers (n =121)
**Confidence related barriers**						
Fear of side effects/side effects from previous dose	302 (24.5)	4 (30.8)	33 (14.2)	2 (8.7)	4 (2.3)	3 (2.5)
Lack of trust in the quality of the vaccine	98 (7.9)	1 (7.7)	-	-	1 (0.6)	1 (0.8)
Lack of trust in the government	67 (5.4)	-	-	-	-	-
**Conspiracy theory, rumour, and misconception**						
It is a mark of the beast	7 (0.6)	-	-	-	-	-
It will lead to infertility	7 (0.6)	-	-	-	-	-
**Complacency related barriers**						
Belief there is no COVID-19	31 (2.5)	-				
I don’t need the vaccine/previous dose(s) taken is sufficient	117 (9.5)	2 (15.4)	114 (48.9)	15 (65.2)	14 (8.1)	22 (18.2)
Loss of vaccine card			8 (3.4)	-	5 (2.9)	-

**Convenience related barriers**						
Long waiting time/busy	77 (6.2)	4 (30.8)	15 (6.4)	4 (17.4)	18 (10.3)	25 (20.7)
Travel out of town/I need to go to where to take second or booster dose	22 (1.8)	-	34 (14.6)	7 (30.4)	33 (19.0)	13 (10.7)
Due to sickness	19 (1.5)	-	3 (1.3)	-	2 (1.2)	3 (2.5)
Lack of access	45 (3.7)	-	16 (6.9)	-	2 (1.2)	1 (0.8)
**Information related barriers**						
Not aware of the COVID-19 vaccine/second or booster dose	88 (7.1)	-	13 (5.6)	-	47 (27.0)	6 (5.0)
**Pregnancy related factors**						
Due to pregnancy	245 (19.9)	-	39 (16.7)	1 (4.4)	6 (3.5)	2 (1.7)
Due to breastfeeding	108 (8.8)	1 (7.7)	3 (1.3)	-		
**Socio-cultural and political related factors**						
It is against my religious belief	37 (3.0)	-	-	-	-	-
Husband did not give permission	9 (0.73)	-	-	-	-	-

Not a beneficiary of political interventions (Cash Transfer policy, government empowerment programme)	10 (0.8)		1 (0.4)	-	-	-
Gender discrimination	1 (0.1)	-	-	-	-	-
**Health systems factor**						
Vaccine is not available	10 (0.8)	1 (7.7)	30 (12.9)	2 (8.7)	45 (25.9)	43 (35.5)
**Others**	14 (1.1)	-	2 (0.9)	-	-	1 (0.8)
