# Supplementary material for: Exploring COVID-19 pandemic perceptions and vaccine uptake among community members and primary healthcare workers in Nigeria: A mixed methods study
Source: PLoS One. 2026 Mar 11;21(3):e0310437. doi: 10.1371/journal.pone.0310437 (PMC12978461; doi:10.1371/journal.pone.0310437)
Supplement: S3 Table — (PDF) [file pone.0310437.s004.pdf]

Characteristics	Total	Community members/patients (N= 1769)	Healthcare workers (N=396)
	N (%)	N (%)	N (%)
**If NO to receiving any dose COVID-19 vaccine (N = 1247)**			
**Do you believe in vaccine safety?**			
Yes	1183 (94.9)	1171 (94.9)	12 (92.3)
No	64 (5.1)	63 (5.1)	1 (7.7)
**Do you believe in vaccine efficacy?**			
Yes	1191 (95.5)	1179 (95.5)	12 (92.3)
No	56 (4.5)	55 (4.5)	1 (7.7)
**If YES to receiving any dose COVID-19 vaccine (N = 1349)**			
**Before the covid-19 pandemic, did you believe in vaccine safety?**			
Yes	1324 (98.2)	945 (97.8)	379 (99.0)
No	25 (1.8)	21 (2.2)	4 (1.0)

**Before the covid-19 pandemic, did you believe in vaccine efficacy?**			
Yes	1326 (98.3)	949 (98.2)	377 (98.4)
No	23 (1.7)	17 (1.8)	6 (1.6)
**Based on your experience with the covid-19 vaccination, do you believe in vaccine safety?**			
Yes	1338 (99.2)	957 (99.1)	381 (99.5)
No	11 (0.8)	9 (0.9)	2 (0.5)
**Based on your experience with the covid-19 vaccination, do you believe in vaccine efficacy?**			
Yes	1335 (99.0)	954 (98.8)	381 (99.5)
No	14 (1.0)	12 (1.2)	2 (0.5)
**Based on your experience with the covid-19 vaccination will you recommend covid-19 vaccine to someone else?**			
Yes	1330 (98.6)	949 (98.2)	381 (99.5)

No	19 (1.4)	17 (1.8)	2 (0.5)
**Based on your experience with the covid-19 vaccination will you allow your child to take routine available vaccines against common childhood illnesses?**			
Yes	1340 (99.33)	960 (99.4)	380 (99.2)
No	9 (0.67)	6 (0.6)	3 (0.8)
**Based on your experience with the covid-19 vaccination are you willing to take vaccine against disease like hepatitis infection?**			
Yes	1316 (97.8)	938 (97.4)	378 (98.7)
No	30 (2.2)	25 (2.6)	5 (1.3)
**Based on your experience with the covid-19 vaccination are you willing to take vaccine against human papilloma virus?**			
Yes	1309 (97.0)	932 (96.5)	377 (98.4)
No	40 (3.0)	34 (3.5)	6 (1.6)

**Based on your experience with the covid-19 vaccination are you willing to take a vaccine in the future in case of outbreak similar to covid-19 pandemic?**			
Yes	1332 (98.7)	951 (98.4)	381 (99.5)
No	17 (1.3)	15 (1.6)	2 (0.5)
