# Supplementary material for: Exploring COVID-19 pandemic perceptions and vaccine uptake among community members and primary healthcare workers in Nigeria: A mixed methods study
Source: PLoS One. 2026 Mar 11;21(3):e0310437. doi: 10.1371/journal.pone.0310437 (PMC12978461; doi:10.1371/journal.pone.0310437)
Supplement: S1 Text — (PDF) [file pone.0310437.s005.pdf]

We will be conducting interviews with healthcare providers who work in primary health facilities that offer routine immunization and COVID-19 immunization services

1. Clinical context

- Can you tell me about a typical immunization clinic (routine immunization) in your setting?
    - Probe: What sort of activities are done? How many children do you see on average?
    - Probe frequency of clinics; clinic duration
    - Probe preparation done prior to the clinic
- If the facility provides COVID-19 immunization services:
    - Probe: What sort of activities are done? What role does the participant perform? How many recipients do you attend to on average per day?
    - Probe frequency of clinics, clinic hours
    - Probe preparation done prior to the clinic (COVID-19 vaccination)
    - If not, probe why it does not offer COVID-19 immunization services
    - What are the processes involved to receive COVID-19 vaccine
    - What is working well with COVID-19 immunization delivery in Nigeria
    - What is not working well with COVID-19 immunization delivery in Nigeria

2. Perception of integrated COVID-19/routine immunization deliveries
    a. What is your opinion of providing COVID-19 immunization with other routine immunization services in your facility as recommended by the NPHCDA?
    b. Does it increase coverage for COVID-19 vaccine? How?
    c. How do healthcare providers consider that approach? Does it help your work? How?
    d. NPHCDA also recommends screening for hypertension and diabetes alongside COVID-19 vaccination. To what extent this is implemented at your facility? How is this approached perceived by the community members.

3. Existing challenges with routine immunization services
    a. What is working well with routine immunization delivery in Nigeria

    b. What is not working well with routine immunization delivery in Nigeria

4. Impact of COVID-19 immunization services on routine immunization service
    a. In what ways has COVID-19 immunization programme affected routine immunization delivery in your facility?
        i. Probe positive impacts if any
        ii. Probe negative impact if any
        iii. Has COVID-19 affected people's perception of vaccines generally in Nigeria? How
    b. What effects does routine immunization have on COVID-19 immunization programme?
        i. Tell me more, can you give examples?
5. COVID-19 vaccine recipients
    a. Have you received at least a dose of the COVID-19 vaccine before? If yes share your experience (registration, verification, and actual inoculation)
        i. Probe motivation for acceptability or hesitancy
            1. Confidence in vaccine
            2. Complacency
            3. Convenience
        ii. Comment on the steps involved to get vaccinated. Were the steps necessary? Why?
        iii. Experiences post-vaccination, side effects if any? How were they managed?
        iv. Were the side effects expected? How?
        v. Perceived difference between COVID-19 immunization services and RI
        vi. Challenges encountered
        vii. Why do some people receive the COVID-19 vaccine?
        viii. Why are other people not willing to accept the vaccine?
6. Do you have any other things to tell me?
7. Thank you for your time
