# Supplementary material for: Exploring COVID-19 pandemic perceptions and vaccine uptake among community members and primary healthcare workers in Nigeria: A mixed methods study
Source: PLoS One. 2026 Mar 11;21(3):e0310437. doi: 10.1371/journal.pone.0310437 (PMC12978461; doi:10.1371/journal.pone.0310437)
Supplement: S2 Text — (PDF) [file pone.0310437.s006.pdf]

We will be conducting interviews with non-healthcare providers who present in primary health facilities

1. Getting to know the participants

- Tell me about yourself: where you live, work you do, marital status, religion and tribe

2. Tell me your perception of immunization generally? Why
    a. What vaccine(s) have you received in your adulthood? Why?
    b. Do you take your children for immunization? Why
    c. Many under-five children are not fully immunized in Nigeria. What could be responsible for this?
    d. Any other challenges regarding immunization programme for children in Nigeria?
    e. What is working well with the ways routine immunization for children is delivered in Nigeria?
    f. What is not working well with the children's routine immunization?

3. COVID-19 vaccination experience
    a. Have you received at least a dose of the COVID-19 vaccine before? If yes, when and how many doses have you received? Share your experience (registration, verification, and actual inoculation), and how satisfied were you with the entire process?
        i. Probe motivation for acceptability or hesitancy
        ii. Why do some people receive the COVID-19 vaccine?
        iii. Why are other people not willing to accept the vaccine?
        iv. Comment on the steps involved to get vaccinated. Were the steps necessary? Why?
        v. What were your experiences post-vaccination, side effects if any? How were they managed? Were the side effects expected? How?
        vi. What is not working well with COVID-19 immunization delivery in Nigeria

vii. What do you consider to be working well with COVID-19 immunization in Nigeria

4. Impact of COVID-19 immunization services on routine immunization service
    a. Of the challenges you discussed so far, which ones are peculiar to:
        i. Routine immunization? How
        ii. COVID-19 vaccine programme? How?
    b. Based on your opinion and experiences, in what ways has the COVID-19 immunization programme affected immunization for children in your setting?
        i. Probe negative impact if any
        ii. Probe positive impacts if any
        iii. Has COVID-19 immunization programme shaped how people perceive vaccines generally?
    c. We have talked about effects of COVID-19 vaccine on routine immunization. Now what effects does routine immunization have on COVID-19 immunization programme?
        i. Tell me more, can you give examples?
