# Supplementary material for: Exploring COVID-19 pandemic perceptions and vaccine uptake among community members and primary healthcare workers in Nigeria: A mixed methods study
Source: PLoS One. 2026 Mar 11;21(3):e0310437. doi: 10.1371/journal.pone.0310437 (PMC12978461; doi:10.1371/journal.pone.0310437)
Supplement: Attachment — Submitted filename: Review report COVID.docx [file pone.0310437.s010.docx]

The study explored COVID-19 pandemic perceptions and vaccine uptake among community members and primary healthcare workers (HCWs) in Nigeria. 43.4% of community members and 96.7% of HCWs received at least one dose of the COVID-19 vaccine. 27.8% of community members and 22.0% of HCWs believed in "African immunity" to COVID-19. The author should address the following points……

1. The study primarily focused on community members and healthcare workers (HCWs) at primary healthcare facilities, potentially overlooking insights from individuals who were not engaged with these facilities. Relying on self-reported vaccine uptake and perceptions may have led to inaccurate results due to memory gaps and the tendency to give socially acceptable answers.
2. While household wealth index and government employment were analyzed, factors like transportation challenges, internet access (for misinformation) and local economic impact of COVID-19 should explored.
3. Only 14 healthcare providers and 16 community members were interviewed, which might not fully capture the diverse range of opinions and experiences in a country as large as Nigeria.
4. The study was conducted in only three Nigerian states (Oyo, Lagos, and Jigawa), which may not fully represent national trends in vaccine hesitancy and uptake.
5. The qualitative analysis did not extensively compare the perceptions across different regions, ethnic groups, or social classes in Nigeria.
6. The study integrates qualitative and quantitative findings, but there are gaps in both analyses. A mixed-methods approach was useful, but the study could have strengthened the correlation by expanding the qualitative sample size for greater diversity.
7. Although spousal approval and pregnancy-related concerns were identified, a deeper gender-based analysis was lacking to explore how social roles and expectations influenced vaccine decision-making
8. The study did not extensively explore behavioral or psychological factors influencing vaccine uptake beyond demographics and basic perceptions. More detailed variables, such as media influence, trust in healthcare institutions, or personal experiences with previous vaccinations, were not deeply analyzed. Including these parameters will expand the scope of variables in enhancing quantitative analysis.
9. In sampling technique indicate whether the study used random sampling, convenience sampling or stratified sampling.
10. In sampling technique indicate whether the study used random sampling, convenience sampling, or stratified sampling like Multivariable logistic regression, Chi-square test, T-tests or ANOVA
