# Supplementary material for: Exploring COVID-19 pandemic perceptions and vaccine uptake among community members and primary healthcare workers in Nigeria: A mixed methods study
Source: PLoS One. 2026 Mar 11;21(3):e0310437. doi: 10.1371/journal.pone.0310437 (PMC12978461; doi:10.1371/journal.pone.0310437)
Supplement: Attachment — Submitted filename: Response to Reviewers.docx [file pone.0310437.s011.docx]

**Reviewer 1**

Introduction:

Provides a good background to the study in line with what the situation was at the time the data was collected. However, now there are many similar studies that have since been published and it would be desirable to include some of the recent studies.

*We thank the reviewer for this helpful comment and for acknowledging the relevance of our introduction to the context in which the data were collected. We agree that since the time of data collection, several additional studies on COVID-19 vaccine uptake have been published. In response, we have updated the Introduction to acknowledge the evolving post-pandemic vaccination landscape briefly. Specifically, we now note that as of 2025, approximately 51.8% of the eligible population in Africa has been vaccinated, and Nigeria currently falls within the 40–69% coverage category, according to recent data from the Africa CDC. This has now been added* ***to page 4, paragraph 1, lines 8-11.***

*We have also reviewed recent literature and included references to new studies where appropriate, ensuring our framing remains current and contextually grounded.*

Methods and Results:

Appropriately described and implemented as stated.

The sample size stated on page 5-6 was estimated at 558 of the three states, and on page 10 the sample size reported is 2602. Need clarification in the way this statement is worded, was 558 for each of the 3 State or what?

*Thank you for this valuable observation and the opportunity to clarify. The estimated sample size of 558 referenced in the Methods section refers to the total minimum sample size for the three states combined, not per state. This figure was based on a sample size calculation using a single proportion formula, which yielded an estimated sample size of 186 participants per state, adjusted for a 10% non-response rate. The 558 was therefore the combined target sample size across Lagos, Oyo, and Jigawa States.*

*However, during data collection, we employed convenience sampling at selected primary healthcare facilities. This approach, along with unexpectedly high participant turnout particularly among community members allowed us to collect a larger-than-expected sample of 2602 participants across all states. This enriched the study’s power and provided greater robustness for our analyses. We have now revised the Methods section of the manuscript* ***on page 7, paragraph 1, lines 6-10*** *to clearly reflect this and prevent misinterpretation.*

A very high number of female respondents (83.9%) – why? And what impact did this have on the overall results/study outcome?

*Thank you for this important observation. The high proportion of female respondents (83.9%) among community members is largely attributable to the design of the larger parent study, the COVID-19 Vaccine Programme Delivery in Nigeria, which examined the delivery and intersection of COVID-19 vaccination and routine immunization programmes in primary health facilities. As a key component of routine immunization services in Nigeria, mothers and female caregivers are almost exclusively the ones who accompany children to health facilities for vaccination appointments. This pattern is culturally normative and well-documented in the Nigerian primary healthcare context, where male caregivers are rarely present during immunization visits. Given that data collection was conducted in outpatient and immunization clinics, often on routine immunization days, it is unsurprising that the majority of participants present and eligible for inclusion were women.*

*In terms of impact, the gender distribution reflects actual service utilization patterns and does not compromise the study’s validity. Rather than introducing bias, it provides a representation of the target population most engaged with immunization efforts. Nonetheless, gender was included as an explanatory variable in our quantitative analysis, and we found that being male was significantly associated with higher odds of COVID-19 vaccine uptake among community members. This was explicitly discussed in the Results and Discussion sections.*

Even in qualitative respondents, majority were women, why?

*Thank you for this thoughtful question. The response to the comment is the same as the previous question.*

*Additionally, our qualitative sampling aimed for maximum variation in vaccine uptake (none, one, or two doses) rather than strict gender balance. Since women constituted the majority of those attending the health facilities during data collection, most information-rich cases were among female participants.*

Page 16, vaccine uptake was lower among community members who practiced Christianity. Why?

*We sincerely thank the reviewer for this important observation. As seen in Table 3, religion was not significantly associated with vaccine uptake in the crude model, but became statistically significant in the adjusted model (AOR = 0.73; 95% CI: 0.55–0.97; p = 0.032), indicating lower odds of vaccination among Christians compared to Muslims. This shift reflects the effect of confounding variables such as age, employment status, sex, and prior COVID-19 exposure, which were adjusted for in the multivariable model. The adjusted result suggests that after accounting for these other factors, Christian community members were less likely to report having received the COVID-19 vaccine. While our study did not explore the underlying reasons for this difference, it may reflect denominational variations in trust, vaccine messaging, or leadership influence. Prior studies and anecdotal evidence have shown that faith-based institutions can play both supportive and limiting roles in vaccine acceptance, depending on local contexts.* ***We have acknowledged this in the revised discussion on pages 25-28 and highlighted the need for further research into the role of religious affiliation and leadership in shaping vaccine decisions.***

Discussion:

1). Expound on your findings in the discussion section by helping readers understand why some of the results came out as they did:

• Examples:

o Why being male was associated with better/higher.

o Being of Christianity faith was associated with low uptake.

*Thank you for this valuable observation. We have now expanded the discussion to help readers better understand why certain associations appeared in our results—such as why being a Christian community member was linked to low uptake* ***on page 26, paragraph 2****—and why being male was linked to higher uptake* ***on page 26, paragraph 3.***

*We have expanded on the finding that male community members were significantly more likely to be vaccinated against COVID-19 than female community members. This association probably reflects a mix of structural, social, and cultural factors. In many parts of Nigeria, men tend to have greater autonomy in health-related decision-making (especially regarding vaccination) and better access to health information, which can promote timely vaccination. Women, as seen among study participants, often encounter barriers such as needing spousal consent and limited mobility due to childcare responsibilities or cultural norms. These factors may decrease their likelihood of seeking vaccination, even when it is available.*

2). The finding of faith leaders in Ethiopia associated with low uptake (page 26 second paragraph) contradicts with last sentence in the discussion (page 27 – 28) from the same reference/citation. The authors need to make this clear for the readers. Which one is which? What is true from the reference/citation?

*We thank the reviewer for this astute observation and agree that the reference to the Ethiopia study requires clarification. The qualitative study conducted in Addis Ababa indeed emphasized that faith leaders acted as key allies in promoting vaccine confidence and uptake, not directly linking religious affiliation (Christianity) to lower vaccination. To address this, we have revised the discussion* ***on the page 26, paragraph 2*** *to:*

*Clarify the association between Christianity and lower vaccine uptake*

*Acknowledge mixed findings in the broader literature regarding religion and vaccine acceptance.*

3). It is helpful to also include studies that are contradictory to some of your findings or those that are inclusive and provide a synthesis of how your study fits into the big picture when you integrate such. It helps strengthen the study but also provides a springboard for further research discussions and research.

*Thank you for this valuable observation. We have now expanded the discussion to include studies that are contradictory to some of your findings or those that are inclusive*

***Citations are made after the “period” this is wrong. As stated above. This appears throughout the document. Should be corrected.

*Thank you for this valuable observation. We have now corrected this in the manuscript.*

*****Citations are made after the “period” this is wrong. As stated above. This appears throughout the document. Should be corrected.**

**Reviewer 2**

I appreciate the submission and this is an important topic to addressed and subsequenlty being published. However, one of the major concerns arrive from this submisision is the combination of the community and HWC. The community and HWCs are of two various background and experienced differences with COVID-19. There was no justification was why both of these groups were combined into the analysis. If the intention is to do comparison, the data analysis failed to capture this. The strength of this manuscript is in it mixed-methods study, which strengthen the study. Given that the number of community is large, perhaps the authors could focus on just this community and leave the HWCs data for a separate submission.

*We sincerely thank the reviewer for this thoughtful and constructive feedback and for recognizing the importance of our study and the strength of its mixed-methods design. We appreciate the concern regarding the inclusion of both community members and healthcare workers (HCWs) in a single manuscript.*

*We included both healthcare workers and community members in this study to gain a comprehensive understanding of COVID-19 vaccine uptake from both recipients and providers of immunisation services. Quantitative analyses were conducted separately for the two groups to ensure clarity and maintain the distinctiveness of their experiences. For the qualitative component, which contributed a smaller portion of data to the mixed-methods integration, interviews were carried out with both groups, and coding and theme development were performed separately before being synthesised into overarching themes to explore system-wide insights. Understanding the attitudes and uptake behaviour of HCWs alongside that of the general population provides a more holistic view of the contextual factors influencing COVID-19 vaccine acceptance. We recognize the importance of clarity and have now revised the manuscript to add this justification for this approach in the* ***Methods section on page 5, paragraph 2, lines 3-10.***

*Given that the study was designed to include both groups, we believe separating the data at this stage would limit the study’s value. However, we agree that focused analyses of HCW data in future publications may be valuable.*

**Reviewer 3**

The study explored COVID-19 pandemic perceptions and vaccine uptake among community members and primary healthcare workers (HCWs) in Nigeria. 43.4% of community members and 96.7% of HCWs received at least one dose of the COVID-19 vaccine. 27.8% of community members and 22.0% of HCWs believed in "African immunity" to COVID-19. The author should address the following points……

1. The study primarily focused on community members and healthcare workers (HCWs) at primary healthcare facilities, potentially overlooking insights from individuals who were not engaged with these facilities. Relying on self-reported vaccine uptake and perceptions may have led to inaccurate results due to memory gaps and the tendency to give socially acceptable answers.

*Thank you for your insightful feedback. While our study focused on individuals engaging with primary healthcare (PHC) facilities, this level of care represents the most accessible point of contact with the health system for many community members. It serves as a major platform for vaccine delivery in Nigeria. To extend our reach beyond facility walls, we also interviewed community health extension workers (CHEWs) who routinely conduct community outreach activities.*

*At the time of data collection, the COVID-19 vaccination campaign was still ongoing, which minimised recall bias, as vaccination experiences were relatively recent. Moreover, unlike conditions such as HIV or tuberculosis, COVID-19 vaccination in Nigeria was not associated with significant stigma. Participants also did not expect benefits from their responses, reducing the possibility of socially desirable answers. Nevertheless, we cannot completely rule out social desirability bias, and we have acknowledged that in our limitation* ***on page 28, paragraph 3, line 1-5.***

*Overall, the study provides insights into vaccine uptake among individuals actively participating in vaccination programmes and reflects broader community dynamics. Future studies could build on this by investigating uptake among those who did not pursue vaccination through PHC facilities.*

2. While household wealth index and government employment were analyzed, factors like transportation challenges, internet access (for misinformation) and local economic impact of COVID-19 should be explored.

*We included government employment and household wealth as sociodemographic variables in our study. However, transportation challenges and internet access were not directly measured, as we did not anticipate them to be major barriers—only residents of the study Local Government Areas (LGAs) were eligible.*

*Our quantitative tool allowed participants to state reasons for not receiving the vaccine, but none mentioned internet access and transport issues, and these factors did not emerge in our qualitative data.*

*While not a primary focus of this study, we acknowledge the relevance of these factors. Future research will explore the local economic impact of COVID-19 on vaccine uptake.*

3. Only 14 healthcare providers and 16 community members were interviewed, which might not fully capture the diverse range of opinions and experiences in a country as large as Nigeria.

*Thank you for your valuable feedback. Our sample size was determined based on methodological guidance and practical considerations to achieve saturation, as stated* ***on page 9, paragraph 1*** *of the manuscript. Despite this, we recognize that in a country as large and diverse as Nigeria, our sample may not fully represent the full range of possible perspectives. However, we adopted a purposive sampling strategy to enhance diversity and depth of insights. Among healthcare providers, we included individuals from different professional cadres, roles in immunization services, and varying levels of experience. For community members, we applied maximum variation sampling to reflect heterogeneity in vaccine uptake, including participants who had received zero, one, or two doses.*

*We also ensured geographic diversity by selecting participants from local government areas (LGAs) that reflect both urban and rural characteristics in Jigawa and Oyo States.*

*While qualitative studies do not aim for statistical generalizability, our approach was designed to ensure rich, contextually relevant insights that illuminate the complexities of COVID-19 vaccine uptake in Nigeria. We have now acknowledged this limitation in the revised manuscript* ***on page 28, paragraph 3.*** *Future studies could explore a larger and more diverse participant pool to further enrich understanding.*

4. The study was conducted in only three Nigerian states (Oyo, Lagos, and Jigawa), which may not fully represent national trends in vaccine hesitancy and uptake.

*As stated on* ***page 6, paragraph 1****, the selection of Lagos, Oyo, and Jigawa states was based on their COVID-19 burden and performance in both routine and COVID-19 immunization programs during the early phases of the vaccine rollout in Nigeria.*

*Additionally, these states were chosen to provide geographical and socio-cultural diversity, with Lagos and Oyo representing the southern region and Jigawa representing the northern region.*

*While the study was conducted in only three states, the vaccination rates observed in our findings align with previous reports from Nigeria, including those from a national survey. This consistency suggests that our results provide meaningful insights into vaccine hesitancy and uptake trends in the country. We have provided a detailed description of study settings so that our readers can make an informed decision about whether the findings are transferable to their context.*

5. The qualitative analysis did not extensively compare the perceptions across different regions, ethnic groups, or social classes in Nigeria.

*Thank you for your comments, we have taken a further look and made some edits* ***on pages 22-25.***

6. The study integrates qualitative and quantitative findings, but there are gaps in both analyses. A mixed-methods approach was useful, but the study could have strengthened the correlation by expanding the qualitative sample size for greater diversity.

*Thank you for your thoughtful comment. As noted earlier (see response to Comment 3), the sample size for our qualitative study was guided by the principle of data saturation as described by Hennink (2022) and the concept of information power. We conducted 16 interviews with community members and 14 with healthcare providers. No new themes emerged, and the data collected were sufficient to address our study objectives.*

*It is also important to note that our primary focus was on understanding the drivers of current COVID-19 vaccine uptake, rather than exploring perceptions or attitudes broadly. Given this narrower focus, the sample size was sufficient for an in-depth exploration of key themes without necessitating a larger, more representative qualitative sample. While we agree that expanding the qualitative arm could have offered additional perspectives, our approach was appropriate for our research questions. It helped generate relevant insights that complement our quantitative findings.*

7. Although spousal approval and pregnancy-related concerns were identified, a deeper gender-based analysis was lacking to explore how social roles and expectations influenced vaccine decision-making.

*We acknowledged that gender dynamics are crucial in vaccine decision-making, particularly in contexts where spousal approval and pregnancy-related concerns influence health behaviours. While our study identified these as barriers, a deeper gender-based analysis was not the primary focus. However, our qualitative findings highlighted how gender norms influenced vaccination decisions, with some women requiring their spouse's consent before receiving the vaccine. Future research could expand on this by conducting a more detailed gender-based analysis to understand how social roles and expectations impact vaccine acceptance and hesitancy across different population groups.*

8. The study did not extensively explore behavioural or psychological factors influencing vaccine uptake beyond demographics and basic perceptions. More detailed variables, such as media influence, trust in healthcare institutions, or personal experiences with previous vaccinations, were not deeply analyzed. Including these parameters will expand the scope of variables in enhancing quantitative analysis.

*We appreciate this insightful observation. While our study primarily focused on demographic factors and vaccine uptake, we agree that behavioural and psychological factors could significantly shape vaccine decisions. Although these variables were not quantitatively analyzed in this study, elements such as trust in the healthcare system and vaccine safety concerns did emerge in the qualitative findings. However, we acknowledge that a more structured exploration of these behavioural determinants would enhance the depth of future studies. We have noted this valuable point and incorporated it into the recommendation section of the revised manuscript* ***on page 29, paragraph 2****.*

9. In sampling technique indicate whether the study used random sampling, convenience sampling or stratified sampling.

*Thank you for your comment. We have now clearly outlined the sampling techniques used in our study in the manuscript* ***(page 7, paragraph 2*** *for quantitative sampling;* ***page 9, paragraph 1*** *for qualitative sampling). A combination of* *purposive sampling and convenience sampling was used for the study.*

10. In sampling technique indicate whether the study used random sampling, convenience sampling, or stratified sampling like Multivariable logistic regression, Chi-square test, T-tests or ANOVA

*Thank you for your comment. As noted earlier, we have now clearly outlined the sampling techniques used in our study in the manuscript* ***(page 7, paragraph 2*** *for quantitative sampling;* ***page 9, paragraph 1*** *for qualitative sampling).*

*References*

*Hennink M, Kaiser BN. Sample sizes for saturation in qualitative research: a systematic review of empirical tests. Soc Sci Med. 2022;292:114523. https://doi.org/10.1016/J.SOCSCIMED.2021.114523*
