# Supplementary material for: Exploring COVID-19 pandemic perceptions and vaccine uptake among community members and primary healthcare workers in Nigeria: A mixed methods study
Source: PLoS One. 2026 Mar 11;21(3):e0310437. doi: 10.1371/journal.pone.0310437 (PMC12978461; doi:10.1371/journal.pone.0310437)
Supplement: Attachment — Submitted filename: Response_to_Reviewers_auresp_2.docx [file pone.0310437.s012.docx]

**Responses to the Academic Editor**

**1. Grammar and Clarity:**

*We sincerely thank the editor for these helpful comments. We have carefully revised the manuscript to address both points and ensured that the requested changes have been implemented consistently across all relevant sections.*

o A light copyedit pass is still advised to correct minor typographical and grammatical inconsistencies (e.g., “COVID-19 is God’ns punishment”).

*Thank you for this observation. We have thoroughly copyedited the manuscript to correct all typographical and grammatical inconsistencies, including the example noted. A detailed review was carried out to ensure clarity and accuracy throughout the text.*

o Ensure uniform tense and parallel sentence structures, especially in thematic summaries.

*We appreciate this important feedback. We have carefully revised the manuscript to ensure uniform tense and parallel sentence structures across all sections, particularly in the thematic summaries. These changes have improved the overall readability and academic precision of the manuscript.*

**2. Figures and Appendices:**

o Figure 1 (on vaccine hesitancy) should be rechecked for clarity and consistency with textual descriptions. Ensure legends and categories match exactly what is described in the body.

*We thank the reviewer for this observation. Upon review, we found that the previously attached legends and categories included elements from findings that were neither represented in Figure 1 nor described in the textual narrative. To ensure clarity and complete alignment with the text, we have removed these extraneous legends and categories on page 23, paragraph 2. The revised Figure 1 caption now contains only the title, ensuring consistency between the figure and the textual descriptions.*

o Make sure Supplementary Tables and Interview Guides (S1–S3) referenced in the manuscript are uploaded and appropriately linked.

*Thank you for this reminder. The supplementary files (S1–S3 Tables and Interview guides: S1-S2 Texts) have already been uploaded. However, as we submit these current responses, we will confirm once again that the files are correctly uploaded and appropriately linked to ensure there are no issues.*

**3. Reference Formatting:**

o Double-check reference consistency (e.g., spacing, author initials) to ensure alignment with PLOS ONE citation style across all 50+ entries.

*Thank you for this observation. We have carefully reviewed and revised all references to ensure full compliance with the PLOS ONE citation style. Specific corrections were made to references 4, 9, 10, 11, 12, 13, 24, and 49 to address formatting inconsistencies related to author initials, spacing, and punctuation.*

*Additionally, reference 23 has been entirely replaced. The previous reference, which provided a projected population estimate for Lagos State, was outdated and has since been shown to be inaccurate by other sources. We have now replaced it with a more recent reference from the United Nations Environment Programme (UNEP), which reflects the current and more reliable population estimate for Lagos State.*
