# Supplementary material for: Exploring COVID-19 pandemic perceptions and vaccine uptake among community members and primary healthcare workers in Nigeria: A mixed methods study
Source: PLoS One. 2026 Mar 11;21(3):e0310437. doi: 10.1371/journal.pone.0310437 (PMC12978461; doi:10.1371/journal.pone.0310437)
Supplement: Attachment — Submitted filename: Commnets K.A.docx [file pone.0310437.s013.docx]

Title & Authorship (Page 1 & 11):
• Generally fine.
Abstract (Page 12):
• Line 2 (Page 12): healthcare workers' (HCWs) -> healthcare workers (HCWs)' (The apostrophe should come after the acronym).

Response: corrected

• Line 8 (Page 12): fuelled -> fueled (PLOS ONE uses American English spelling).

Response: corrected

• The abstract is well-written and summarizes the key findings effectively.

Response: Thank you

Introduction (Pages 13-15):
• Page 14, Line 1: 66.1% of the eligible population had been vaccinated -> 66.1% of the global eligible population had been vaccinated (Add “global” for clarity).

Response: corrected

• Page 14, Line 10: This emphasizes -> This emphasizes (American English).

Response: corrected

• Page 15, Line 1: complexities inherent in COVID-19 immunization programmes; and to learn -> complexities inherent in COVID-19 immunization programs and to learn (Remove semicolon; American English: “programs”).

Response: corrected

Methods (Pages 15-21):
• Study Design (Page 15):
o Preservationaintain -> preserve and maintain (Typo).

Thank you, this is the tracked changes version. We confirm it is preserve and maintain in the clean version. It now reads as preserve the distinctiveness…

o programmes; -> programs (American English, remove semicolon).
o it exerts influences on-and differs from -> it influences and differs from (Awkward phrasing).

Thank you, it was corrected in the version we sent earlier. But we have checked the current version and confirmed it aligns with the suggestion.

• Study Settings (Page 16):

.o land arcamass -> land area (Typo).

Thank you we have checked the current version and confirmed it aligns with the suggestion.

o largesthighest population, with an estimated at population of18-20million–24.6 million -> largest population, estimated at 24.6 million (Major typo/editing error. The correct figure from the new UNEP source is 24.6M? The text is garbled and must be fixed).

Thank you for your suggestion. The estimate has been updated

o based on their feasibility -> based on feasibility (Remove “their”).

Response: There is no their in the clean version we submitted. We have also checked the current version to ensure alignment with your suggestion.

• Study Population (Page 16):
o healthcare providers involved in immunization afin the selected study sites -> healthcare providers involved in immunization at the selected study sites (Typo).

Response: This is not in the clean version we submitted. Probably the editor reviewed the version in track changes. We have confirmed the current version aligns with the suggestion made. Thank you.

o facilities inof the selected LGAs -> facilities in the selected LGAs (Typo).

Response: This is not in the clean version we submitted. Probably the editor reviewed the version in track changes. We have confirmed the current version aligns with the suggestion made. Thank you.

o were not residents of LGA residents -> were not residents of the LGA (Repetitive).

Response: This is not in the clean version we submitted. Probably the editor reviewed the version in track changes. We have confirmed the current version aligns with the suggestion made. Thank you.

• Quantitative Data Collection (Page 17-18):
o Data waswere collected -> Data were collected (“Data” is plural).

Response: This is not in the clean version we submitted. Probably the editor reviewed the version in track changes. We have confirmed the current version aligns with the suggestion made. Thank you.

o Sociodemographic information assessed were was -> Sociodemographic information assessed was (Subject-verb agreement).

Response: This is not in the clean version we submitted. Probably the editor reviewed the version in track changes. We have confirmed the current version aligns with the suggestion made. Thank you.

o performed forto ensure -> performed to ensure (Typo).

We have confirmed the current version aligns with the suggestion made. Thank you.

o Household Household wealth index -> The household wealth index (Duplicate word).

We have confirmed the current version aligns with the suggestion made. Thank you.

o including the pattern of vaccination, and reasons -> including vaccination patterns, reasons (Awkward insertion).

We have confirmed the current version aligns with the suggestion made. Thank you.

• Qualitative Data Collection (Page 19-20):
o we interviewed 16 individuals (Jigawa: 8, and Oyo: 8), with maximum variation sampling to include those who received zero, 1, and 2 doses. This sentence is repetitive with the one before it. Combine or delete.

The sentence is deleted in the track changes version submitted earlier. We have confirmed the current version aligns with the suggestion made. Thank you.

o included four sections: focused on -> included four sections: (Remove "focused on”).

The sentence is deleted in the track changes version submitted earlier. We have confirmed the current version aligns with the suggestion made. Thank you.

o who have had prior experience -> who had prior experience (Tense).

The sentence is deleted in the track changes version submitted earlier. We have confirmed the current version aligns with the suggestion made. Thank you.

o aware acquainted -> were acquainted (Typo).

The sentence is deleted in the track changes version submitted earlier. We have confirmed the current version aligns with the suggestion made. Thank you.

o KOA wasis -> KOA is (Tense).

Corrected, thank you

o stored-in on a secure cloud -> stored on a secure cloud (Typo).

The sentence is deleted in the track changes version submitted earlier. We have confirmed the current version aligns with the suggestion made. Thank you.

o double-blinded to each other’s coding -> "Double-blinded" is unusual in qualitative research. Simpler: coded the data independently.

Thank you, rephrased for clarity

o which was were then shared withexpanded to -> which were then shared with (Grammar/typo).

The word is deleted in the track changes version submitted earlier. We have confirmed the current version aligns with the suggestion made. Thank you.

• Reflexivity (Page 20-21):
o spoke the same language as them–participants -> spoke the same language as the participants (Awkward).

Thank you, correction made

o sensitivity; and recognizing -> sensitivity and recognized (Punctuation/tense).

Thank you. We have confirmed the current version aligns with the suggestion made.

o shape influence -> influence (Choose one).

Shape was deleted in the track changes version sent earlier. We have confirmed the current version aligns with the suggestion made. Thank you.

o thate potential influence of their professional roles -> the potential influence of their professional roles (Typo).

Corrected, thank you for the suggestion

o affecton their understanding of their–participants’ experiences -> affect their understanding of the participants’ experiences (Typos/awkward phrasing).

Thank you, we confirm the current version aligns with the suggestion.

o AAB, as a male community health physician, might bring a clinical perspective to the analysis; by emphasizing -> AAB, a male community health physician, might bring a clinical perspective to the analysis, emphasizing (Punctuation).

Thank you, we confirm the current version aligns with the suggestion. It now reads: AAB, a male community health physician, might bring a clinical perspective to the analysis by emphasizing individual health behaviours, patient-provider interaction, and the impact of medical misinformation on vaccine acceptance

o including such as healthcare -> such as healthcare ("including such as" is redundant).

Thank you, we confirm the current version aligns with the suggestion.

o as well as and public health interventions into promoting -> and public health interventions to promote (Grammar/typo).

Thank you, we confirm the current version aligns with the suggestion.

o thesedifferingdifferent backgrounds -> these different backgrounds (Redundant).
Results (Pages 21-35):

Thank you, corrected

• Page 21, Participant Description: The majority of the Among community members -> Among community members, the majority (Editing error).

Thank you, we confirm the current version aligns with the suggestion.

• Page 22, Table 1: *Three respondents practise traditional religion -> *Three respondents practice traditional religion (American English).

Corrected, thank you

• Page 24, Table 2: having been tested (undergone testing. -> having been tested. (Editing error).

Thank you, we confirm the current version aligns with the suggestion.

• Page 25, Text: increased with increasing age [AOR = 1.04;...] and [AOR = 1.06;...], respectively, as well as withand previous COVID-19 testing -> increased with increasing age... and with previous COVID-19 testing (Typo/awkward phrasing).

Corrected, thank you

• Page 25, Text: Uptake was lower among those community members who practised Christianity -> Uptake was lower among community members who practiced Christianity (Typo).

Corrected, thank you

• Page 28, Table 5: COVID-19 is God’ns punishment -> COVID-19 is God's punishment (Grammatical error noted by the editor, still present).

Thank you for your comment. We confirm the current version aligns with the suggestion

• Page 31, Table 6: respondents that-who received -> respondents who received (Typo).

Thank you for your comment. We confirm the current version aligns with the suggestion

• Page 32, Qualitative Quotes: “I saw our President; he took his own... So, who am I” -> “I saw our President; he took his own... So, who am I?” (Add closing question mark).

Done, thank you for the suggestion

• Page 33, Fig 1 Caption: The note in parentheses is critical context that should likely be moved into the main text or the figure legend itself to ensure clarity, as per the editor's request.

Thank you for your comment. We have made the suggested changes
• Page 33, Text: these were linked concerns were related to -> these concerns were related to (Repetitive).

Thank you for the suggestion. The phrase now reads: “these were linked to misinformation…”

• Page 33, Text: Participants mentioned expressed-rumours -> Participants expressed rumours OR Participants mentioned rumours (Typo).

Thank you for your comment. We confirm the current version aligns with the suggestion

• Page 33-34, Text: receive the vaccine;- -> receive the vaccine. (Replace semicolon with period).

Changed as advised

o required before seeking vaccinations;- -> required before seeking vaccinations.

Changed as advised

o were recurring challenges;- -> were recurring challenges.

Changed as advised

• Page 34, Text: vaccine is are enough -> vaccines are enough (Subject-verb agreement).

Thank you, this has been rechecked. We confirm subject-verb agreement

• Page 34, Text: unvaccinated and vaccinated respondents–in their -> unvaccinated and vaccinated respondents in their (Remove stray hyphen).

Thank you, we confirm there is no hyphen

• Page 34, Text: increased with COVID-19-to 99.2% -> increased to 99.2% (Editing error).

Thank you, we confirm the current version aligns with the recommendation

Discussion (Pages 35-39):
• Page 35, Line 1: underscored highlighted -> highlighted (Redundant).

Thank you, ‘underscored’ was deleted in the previous version. This version has no ‘underscored’

• Page 35, Line 3: highlighting underscoring persistent ongoing challenges -> highlighting persistent challenges (Redundant).

We have changed underscoring to highlighting and also unconfirmed to spelling mistakes

• Page 35, Line 5: has failed to meet not attained -> has not attained (Redundant).

Thank you, we confirm the current version aligns with the recommendation

• Page 36, Line 3: appeared to served as -> served as (Grammar).

Thank you, corrected

• Page 36, Line 8: vaccination-than Christians -> vaccination than Christians (Typo).

Thank you, the sentence reads as “African Muslims were more likely than Christians to support COVID-19 vaccinations.”

• Page 37, Line 1: have demonstratedshowed higher acceptance -> showed higher acceptance (Redundant).

Thank you, the sentence reads as “Catholics and non-religious groups have demonstrated higher acceptance”

• Page 37, Line 2: AdditionallyFurthermore -> Furthermore (Choose one).

We have furthermore in the manuscript

• Page 37, Line 3: emphasizedhighlighted -> highlighted (Redundant).

Thank you, we have ‘emphasized’ in the manuscript, but we have changed it to ‘highlighted’ as suggested

• Page 37, Line 4: influenced more influenced by -> influenced more by (Repetitive).

Thank you, we have influence more by social and…in the manuscript.

• Page 37, Line 9: mayean facilitatepromote -> may promote (Editing error).

Like many others, this is not in the cleaned version. However, we have changed facilitate to promote to align with the editor suggestion

• Page 37, Line 10: bothas observed in -> as observed in (Typo).

Thank you, resolved

• Page 37, Line 11: the need forrequiring -> requiring (Redundant).

The phrase reads as “the need for consent, limited mobility due to childcare responsibilities…

• Page 37, Line 12: and-limited mobility -> limited mobility (Typo).

We have “limited mobility” in the manuscript

• Page 37, Line 13: FurthermoreAdditionally -> Additionally (Choose one).

We have ‘furthermore’ in the manuscript

• Page 37, Line 13: mayean reduceaffect -> may reduce (Editing error).

We have “may reduce” in the manuscript

• Page 37, Line 18: thatabout the media exaggerated the disease-being exaggerated -> that the media exaggerated the disease (Awkward phrasing).

We have the suggested phrase in the manuscript

• Page 38, Line 1: identifiedfound -> found (Redundant).

Changed

• Page 38, Line 2: includingwith claims -> including claims (Grammar).

Thank you. We have “including claims” in the manuscript

• Page 38, Line 3: have fosteredled-to mistrust -> have fostered mistrust (Awkward).

Thank you, we have fostered mistrust in the manuscript
• Page 38, Line 4: correctrectify -> rectify (Redundant).

Thank you, we have this in the manuscript: “These findings underscore the need for comprehensive strategies to correct misbeliefs and misinformation..”

• Page 38, Line 6: and among their congregations -> This is a new idea. Consider integrating it better: ...religious leaders, who are highly regarded in African communities, to address misconceptions among their congregations and enhance...

Thank you for your suggestion. We confirm the texts in the manuscript align with the suggestion

• Page 38, Line 9: identifiedreported -> identified (Redundant).

Thank you, reported is deleted.

• Page 38, Line 10: particularlyespecially -> particularly (Redundant).

Thank you, we confirm particularly is used

• Page 38, Line 12: revealedprovided -> revealed (Redundant).

Thank you. The phrase reads as: “The qualitative data revealed additional barriers…”

• Page 38, Line 18: indicating athe need -> indicating a need (Typo).

Thank you. The texts read as indicating a need

• Page 39, Line 1: identifiedfound -> identified (Redundant).

Thank you. The statement reads as identified

• Page 39, Line 3: observedsw the influence -> observed the influence (Typo).

Confirmed the suggestion aligns with the text in the manuscript

• Page 39, Line 4: receivetake the vaccine -> receive the vaccine (Choose one).

Confirmed the suggestion aligns with the text in the manuscript. The manuscript reads as “receive the vaccine”

• Page 39, Line 5: receivingtaking the vaccine -> receiving the vaccine (Choose one).

Confirmed the suggestion aligns with the text in the manuscript. The manuscript reads as Among HCWs, receiving the vaccine to set an example for others and fulfil job requirements was particularly evident”

• Page 39, Line 7: and congregations not only -> not only (Remove “and congregations”; it breaks the sentence flow. The point is that leaders promoted measures).

Yes, the suggestion is what we have in the manuscript text: where religious leaders who are highly respected in their communities, not only promoted preventive measures during the pandemic

• Page 39, Line 12: curutilizing a mixed-method approach in our study provided -> utilizing a mixed-methods approach provided OR our mixed-methods approach provided (Typo/awkward phrasing).

Thank you. The phrase reads as “our mixed-method approach provided a unique “

• Page 39, Line 13: understandcomprehend -> understand (Redundant).

Yes, we have “opportunity to understand” in the manuscript

• Page 39, Line 14: futureduring another pandemics -> future pandemics (Editing error).

Yes, we have future pandemics

References (Pages 40-45, 87-93):
• The authors state they have fixed references 4, 9, 10, 11, 12, 13, 24, 49 and replaced 23. This must be verified in the final submitted manuscript file. The reference list in the provided text appears correct, but the final submission must be checked meticulously for PLOS ONE style (e.g., journal abbreviations, use of “et al.”, punctuation, etc.).

Thank you, we have done that again

Response to Reviewers (Page 94-95):
• The responses are professional and address the editor's points adequately.
________________________________________
Summary of Major Recommendations
1. Thorough Copyedit: The manuscript requires a complete, word-by-word copyedit by a native English speaker or professional editing service focused on:
o Grammar and syntax.
o Tense consistency (often shifts between past and present).
o American English spelling (e.g., emphasize, practice (verb), program).
o Removing redundant phrases and awkward constructions.
2. Consistency Check: Ensure all instances of “programme” are changed to “program”. Ensure all demographic data (especially the Lagos population figure) is accurate and consistently reported.
3. Figure 1 Legend: Integrate the explanatory note from the caption (about “other reasons” and “conspiracy theories”) into the main results text for clarity, as this directly addresses the editor's point about aligning the figure with the text.
4. Final Proofread: After implementing all changes, the manuscript must be proofread one final time to catch any remaining errors before submission.
This study makes a valuable contribution. By meticulously addressing these language and presentation issues, the authors will significantly strengthen their manuscript and improve its chances of acceptance.

Thank you for the suggestions. We have gone through and made all necessary corrections
